# Genome sequence of the *Bacteroides fragilis *phage ATCC 51477-B1

**DOI:** 10.1186/1743-422X-5-97

**Published:** 2008-08-18

**Authors:** Shawn A Hawkins, Alice C Layton, Steven Ripp, Dan Williams, Gary S Sayler

**Affiliations:** 1 Department of Biosystems Engineering and Soil Science, The University of Tennessee, Knoxville, TN, USA; 2 Department of Microbiology, The University of Tennessee, Knoxville, TN, USA; 3 The Center for Environmental Biotechnology, The University of Tennessee, Knoxville, TN, USA

## Abstract

The genome of a fecal pollution indicator phage, *Bacteroides fragilis *ATCC 51477-B1, was sequenced and consisted of 44,929 bases with a G+C content of 38.7%. Forty-six putative open reading frames were identified and genes were organized into functional clusters for host specificity, lysis, replication and regulation, and packaging and structural proteins.

## Findings

Bacteriophages infecting *Escherichia coli *and *Bacteroides fragilis *serve as fecal pollution indicators (1). *Bacteroides *phages are more attractive fecal indicators than *E. coli *because *Bacteroides *are more abundant in fecal matter, provide higher host specificity, and are anaerobic and thus less likely to reproduce in aquatic environments than *E. coli *[1-4]. Phage ATCC 700786-B1, infecting *B. fragilis *RYC2056, serves as an ISO standard reference phage, but the host is also susceptible to phages in other animal feces [5,6]. Phage ATCC 51477-B1 infects the host *B. fragilis *HSP40, which is reported to be only susceptible to phages in human feces and surface water polluted with municipal/septic wastewater [7].

A drawback to using *Bacteroides *phages as water quality indicators results from the requirement to cultivate them on an anaerobic host [6]. This can be circumvented with direct phage detection via PCR [8], but assay design is difficult because only one gene for one *Bacteroides *bacteriophage is publically available. The lack of ability to detect *Bacteroides *bacterial phage is startlingly inadequate because the host genera is dominant in the human gut and contains important antibiotic resistant pathogens [3,9]. In this study, the genome of phage ATCC 51477-B1 was sequenced to promote fecal source tracking assay development and aid construction of a *B. fragilis *phage bioreporter [10].

**Figure 1:**
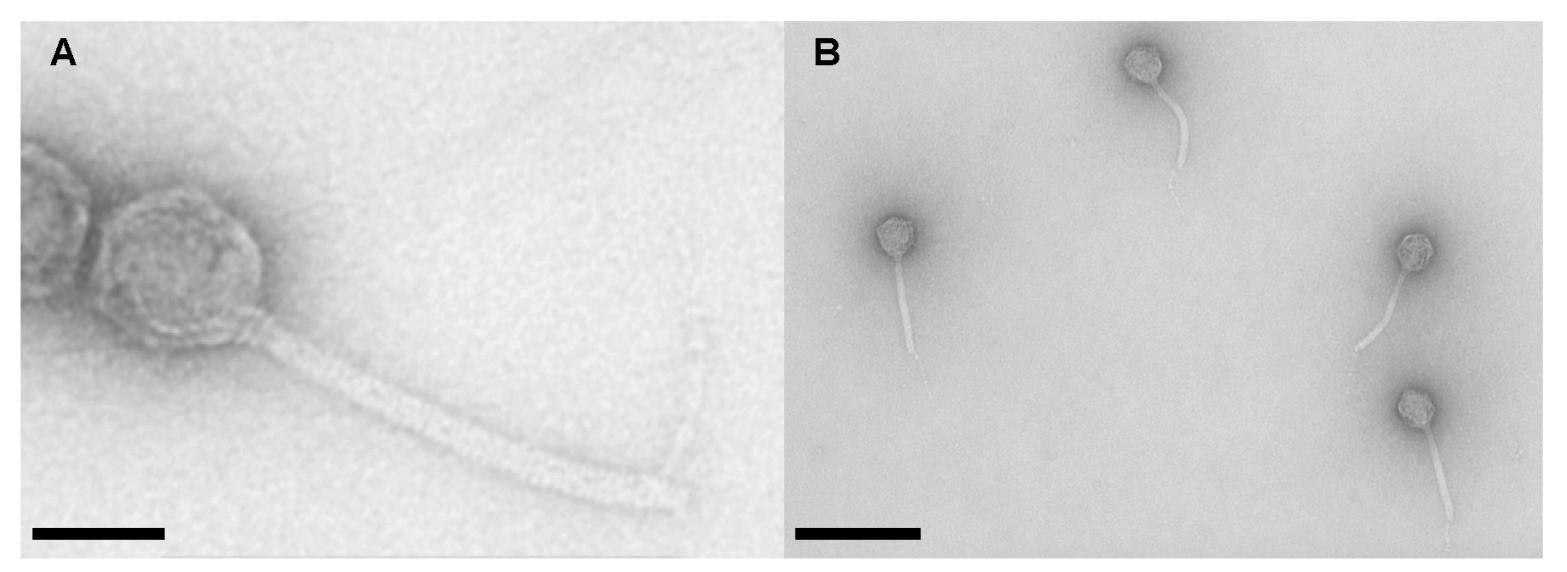
**Transmission electron micrographs of phage ATCC 51477**. (A) Magnified view of a single phage (bar = 50 nm). (B) Four additional phage all displaying tail fibers (bar = 200 nm).

Phage ATCC 51477-B1 was propagated on *B. fragilis *HSP40 (ATCC 51477) at 37°C in anaerobic *Bacteroides *phage recovery medium (BPRM) amended with kanamycin, nalidixic acid, bile salts, and Oxyrase (Oxyrase; Mansfield, OH) [11,12]. Phage were purified and concentrated from lysate using polyethylene glycol [13] and displayed a non-elongated icosohedral head and non-contractile tail consistent with the family Siphoviridae and other *B*. *fragilis *phages isolated from municipal wastewater (Figure 1) [14]. Structural dimensions were similar to *B. fragilis *phage B40-8 with respect to head diameter (60 ± 4.0 μm) and tail length (162 ± 17.0 μm), but the tail diameter was greater (13.4 ± 1.1 μm versus 9.3 ± 0.4 μm) [13].

**Figure 2:**
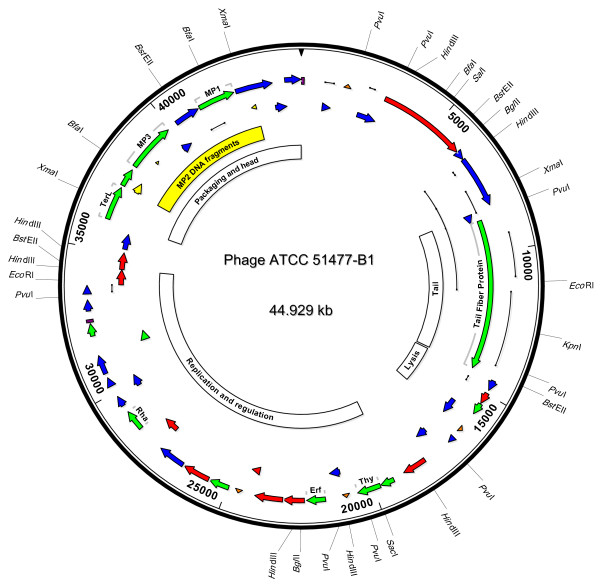
**Phage ATCC 51477-B1 genome map**. The direction and size of 46 putative ORFs are illustrated with arrows. ORFs labeled in green display high similarity to proteins with known function. ORFs labeled in red display high similarity to other putative open reading frames without assigned functions. ORFs labeled in blue have low similarity (< 0.1) to other putative open reading frames. Sequences with high DNA similarity to the phage B40-8 structural proteins are shown in yellow. Putative *B. fragilis *promoters are shown as inverted orange triangles, the location of repeats are shown as purple boxes, and polymorphisms are indicated with black lines.

Phage DNA was extracted with Lambda minipreps (Promega, Madison, WI) and digested with *Hin*dIII. Several *Hin*dIII restriction fragments were cloned into pUC19 [15] and sequenced using primer walking. PCR reactions bridging the cloned *Hin*dIII restriction fragments were cloned and sequenced to confirm fragment order. Non-redundant areas of the genome that were difficult to clone were directly sequenced. In total, dideoxynucleotide sequencing provided 2× coverage except at the 5' and 3' ends. Confirmation of sequence data was sought by GS FLX pyrosequencing (MWG Biotech, Inc., High Point, NC) which produced 23,263 reads, 58,730 base calls, and assembly of 62 contigs using the GS *De Novo *Assembler. Thirty eight of the contigs with more than 3 reads were co-assembled with the dideoxynucleotide sequences using DNASTAR Lasergene 7.1 software suite (DNASTAR, Inc., Madison, WI). A total of 21,935 pyrosequencing reads in 27 contigs were maintained in the final assembly which aligned at 91% similarity for 100× coverage of the phage genome, including the 5' and 3' ends. The final assembled genome was 44,929 bp with a 38.7% G+C content (Figure 2).

Open reading frames (ORFs) were identified using GeneMark [16,17] and the NCBI ORF Finder and examined for known protein functions, structures, and motifs using a conserved domain database [18] (Additional file 1, Figure 2). Ten of the 46 putative ORFs contained amino acid sequences with predictable functions or motifs (Additional file 1). The 5' ends of three ORFs (ORF39, ORF40, and ORF43) displayed translated similarity to previously published N-terminal amino acid sequences for *B. fragilis *phage B40-8 head (MP1 and MP3) and tail (MP2) proteins at 100, 80, and 90% similarity, respectively [13]. The B40-8 MP2 gene (the only *B. fragilis *phage gene present in GenBank), was present in ATCC 51477-B1 but contained a 119 bp insertion and was split into three misarranged sections, one of which was inverted (Figure 2). This suggested the putative ATCC 51477-B1 tail protein was chimeric with respect to B40-8.

The ATCC 51477-B1 genome contained gene functional clusters for host specificity (tail fiber), lysis, replication and regulation, and packaging and structural proteins (Figure 2) [19]. The host specificity region contained ORF7 with translated similarity to the tail fiber of *Enterococcus *phage phiEF24C (Additional file 1) and a large size (1,897 amino acids) suggesting it may be a tape measure gene [20]. A putative M-15 type peptidase (ORF10) was the only gene clearly linked with the lysis region. The replication and regulation region included phage anti-repressors (ORF22 and ORF26), DNA modifying enzymes (ORF15 and ORF16), and replication proteins (ORF22, ORF31, and ORF32). Within the packaging and structural cluster, an ORF with similarity to the TerL protein was identified (ORF 38), as well as ORFs with N-terminal sequences similar to the three phage B40-8 structural proteins previously mentioned (ORF39, ORF40, and ORF43) [13].

Four potential promoters were identified on the positive strand of the genome based on similarity to promoters found in *B. fragilis *[21]. Two were in the regulation and replication region, 5' of ORFs 17 and 22. The other two potential promoters were near the beginning of the genome, 5' of ORF2, and in the lysis region, 5' of ORF12. A tandem repeats finder [22] revealed a 23 bp repeat within an 83 bp segment having two perfect and two degenerate repeats in the replication and regulation region. Another 96 bp perfect repeat sequence was identified at the beginning of the phage genome and again at the end of the replication and regulation region (Figure 2).

Eight polymorphic regions occurring within ORFs and displaying sequence variability from 4% to 13% were identified with the extensive pyrosequencing data. These regions ranged in size from 200 bp to greater than 1,000 bp. ORF7, the putative tail fiber protein, displayed the most polymorphisms, including a pyrosequencing contig with a deletion relative to the final assembly. Tail fiber variability modifies the phage host range [23] and may, for this phage, reflect the antigenic variability of *B. fragilis *surface components [21,24].

The sequence for *B. fragilis *phage ATCC 51477-B1 was deposited in GenBank with accession number FJ008913.

## Competing interests

The authors declare that they have no competing interests.

## Authors' contributions

SAH cloned and sequenced the genome by primer walking, assisted in the pyrosequence data analysis and annotation, and drafted the manuscript. ACL analyzed pyrosequencing data, annotated the genome, and assisted in drafting the manuscript. SR organized the study and provided final editing for the manuscript. DW provided genome cloning. GSS participated in the study design and coordination. All authors read and approved the final manuscript.

## Supplementary Material

Additional file 1 Phage ATCC 51477-B1 genome organization. Click here for file
