# Supplementary material for: Genome sequence of the Bacteroides fragilis phage ATCC 51477-B1
Source: Virol J. 2008 Aug 18;5:97. doi: 10.1186/1743-422X-5-97 (PMC2535602; doi:10.1186/1743-422X-5-97)
Supplement: Additional file 1 — Phage ATCC 51477-B1 genome organization. [file 1743-422X-5-97-S1.pdf]

Open Reading Frames				Protein Mass, Function and Database Matches				
No.	Coordinates		Size (aa)	a. a. (kDa)	Predicted function	Gene Product	Identical a.a.	E-value
	Start	End						
1	825	1151	108	12309	Hypothetical			
2	2248	3000	250	28723	Hypothetical			
3	3012	6152	1046	117229	Conserved phage	Hypothetical protein ALIPUT_01650 (*Alistipes putredinis* DSM 17216) (ZP_02425503)	112(313)	2e-47
						Hypothetical protein PHG11b_40 (*Flavobacterium* phage 11b) (YP_112516)	67(271)	8e-05
4	6152	6478	108	12638	Hypothetical			
5	6492	8324	610	69592	Hypothetical			
6	8384	8731	115	12984	Hypothetical			
7	8725	14417	1897	207559	Putative tail fiber	Hypothetical protein BACCAC_00720 (*Bacteroides caccae* ATCC 43185) (ZP_01959124)	48(118)	1e-13
						Putative tail fiber protein (*Enterococcus* phage phiEF24C) (YP_001504140)	53 (165)	2e-10
8	14509	15372	119	13525				
9	14977	15372	131	15187	Conserved protein	Hypothetical protein BACOVA_03950 (*Bacteroides ovatus* ATCC 8483) (ZP_02066948)	69(128)	3e-28
10	15396	15794	132	14913		Hypothetical protein BF2347 (*Bacteroides fragilis* YCH46) (YP_099631)	78(130)	2e-35
					Putative peptidase	Peptidase M15A (*Pseudomonas putida* W619) (ZP_01641347)	50(129)	2e-17
						pfam08291, Peptidase_M15		1e-25
						COG3108, Uncharacterized conserved protein in bacteria		5e-04
11	15801	16406	201	22982	Conserved protein	Hypothetical protein BACSTE_02147 (*Bacteroides stercoris* ATCC 43183) (ZP_02435894)	58(193)	7e-10
12	16817	17062	81	9148	Hypothetical			
13	17380	17796	138	15984	Hypothetical			
14	18042	18878	278	32543	Conserved protein	Hypothetical protein BACCAC_02353 (*Bacteroides caccae* ATCC 43185) (ZP_01960735)	112(301)	1e-42
						Pfam07083, DUF1351-phage unknown function		1e-07
15	19275	19757	160	18255	Putative DNA N-6-	Hypothetical protein BF2807 (*Bacteroides fragilis* YCH46) (YP_100091)	97(159)	4e-56
					adenine	gp44 (*Burkholderia* phage Bcep176) (YP_355379)	61(149)	1e-26
					methyltransferase (Dam)	pfam05869, Dam, DNA N-6-adenine-methyltransferase		7e-10
16	19768	20565	265	30710	Thymidylate synthase	Thymidylate synthase (*Bacillus coagulans* 36D1) (ZP_01697311)	173(266)	5e-100
					(ThyA)	Hypothetical protein BACUNI_01649 (*Bacteroides uniformis* ATCC 8492) (ZP_02070231)	167(265)	4e-96
						Pfam00303, Thymidylate synthase		7e-103
						COG0207, ThyA, Thymidylate synthase		2e-105
17	21009	21404	131	14730	Hypothetical			
18	21658	22290	210	23608	DNA single-stranded	Essential recombination function protein (*Clostridium novyi* NT) (YP_878265)	74(141)	3e-33
					annealing protein	Essential recombination function protein Erf (*Enterobacteria* phage HK022) (NP_037690)	72(146)	3e-29
						Pfam04404, ERF superfamily		7e-15
19	22365	23078	237	28241	Conserved protein	Hypothetical protein EUBDOL_01513 (*Eubacterium dolichum* DSM3991) (ZP_02077716)	92(223)	7e-40
20	23091	24035	314	358870	Conserved protein	Hypothetical protein BVU_2860 (*Bacteroides vulgatus* ATCC 8482) (YP_001300128)	116(306)	6e-48
						Gp51 (*Mycobacterium* phage Pipefish) (YP_655328)	47(152)	0.002
21	24038	24340	100	11428	Conserved protein	Bacteriophage-related protein (*Oceanicola batsensis* HTCC2597) (ZP_01000936)	26(97)	0.099
22	24889	25626	227	25736	Phage antirepressor	gp36 (*Listeria* phage A006) (NP_469426)	109(226)	7e-54
						COG3617, Prophage antirepressor (transcription)		3e-24
						pfam03374, ANT Phage antirepressor protein		9e-32
23	25668	26591	307	35537	Conserved protein	Hypothetical protein M23134_05729 (*Microscilla marina* ATCC 23134) (ZP_01689133)	73(277)	3e-10
24	26651	27574	307	35813	Hypothetical			
25	27579	28157	192	22025	Conserved protein	blr4528 (*Bradyrhizobium japonicum* USDA 110) (NP_771168)	41(150)	0.024
26	28513	29217	234	27004	Conserved regulatory	Uncharacterized phage-encoded protein (*Desulfovibrio desulfuricans* G20) (YP_388229)	68(132)	9e-31
						Antirepressor, putative (*Enterococcus faecalis* V583) (NP_815702)	59(109)	4e-24
						Pfam09669, Phage_pRha, Phage regulatory protein Rha (Phage_pRha)		7e-24
						COG3646, Uncharacterized phage-encoded protein		1e-15
27	29473	29832	119	140000	Hypothetical			
28	29792	30229	145	16870	Hypothetical			
29	30226	30540	104	12357	Hypothetical			
30	30689	31339	216	25197	Hypothetical			
31	31326	31757	143	17340	HNH endonuclease	SLT orf 104b-like protein (*Staphylococcus* phage phi 12) (NP_803334)	27(75)	0.053
32	32017	32442	141	15772	SSB_OBF (ssDNA-binding protein)	Ssb (*Escherichia coli* APEC 01) (YP_001481169) Protein family HMM PF00436	58(143)	8e-23
						Pfam00436 SSB, Single-strand binding protein family		4e-25
33	32864	33238	124	14439	Hypothetical			
34	33433	33723	96	11400	Hypothetical			
35	33747	34349	200	23244	Conserved protein	Hypothetical protein BACCAC_01178 (*Bacteroides caccae* ATCC 43185) (ZP_01959570)	59(180)	2e-13
36	34356	35021	221	25636	Conserved protein	Hypothetical protein ORF022 (*Pseudomonas* phage PA11) (YP_001294615)	30(62)	8e-10
37	35166	35768	200	23490	Hypothetical			
38	36986	37279	397	45494	Terminase Large Subunit	Putative terminase large subunit (*Lactococcus* phage ul36) (NP_663671)	115(342)	5e-36
						COG5410		9e-09
39	37365	38003	212	222725	Major tail protein	Tail major protein 2 (Bacteriophage B40-8) (AF074719)	133(135)	4e-71
40	38151	39884	577	67508	Putative Capsid	Matches major protein 3 (Bacteriophage B40-8) N-terminal amino acid sequence	16(20)	
41	39847	40245	132	15579	Hypothetical			
42	40245	41162	305	33557	Hypothetical			
43	41213	42529	438	48419	Putative Capsid	Matches major protein 1 (Bacteriophage B40-8) N-terminal amino acid sequence	20(20)	
44	42598	43911	437	498896	Hypothetical			
45	43908	44360	150	17605	Hypothetical			
46	44350	44919	189	22029	Hypothetical			
